# Electrical Conductivity and Antibacterial Activity of Woven Fabrics through Quercetin-Assisted Thermal Reduction of a Graphene Oxide Coating

**DOI:** 10.3390/ma16227184

**Published:** 2023-11-16

**Authors:** Mariia Svyntkivska, Tomasz Makowski, Dorota Kregiel, Ewa Piorkowska

**Affiliations:** 1 Centre of Molecular and Macromolecular Studies, Polish Academy of Sciences, Sienkiewicza 112, 90-363 Lodz, Poland; tomasz.makowski@cbmm.lodz.pl; 2 Department of Environmental Biotechnology, Faculty of Biotechnology and Food Sciences, Lodz University of Technology, Wolczanska 171/173, 90-924 Lodz, Poland; dorota.kregiel@p.lodz.pl

**Keywords:** electroconductive fabric, reduced graphene oxide, antibacterial activity, quercetin

## Abstract

Cotton and poly(ethylene terephthalate) (PET) woven fabrics were coated with graphene oxide (GO) using a padding method and the GO deposited on the fiber surfaces was thermally reduced to impart electrical conductivity to the fabrics. To assist the thermal reduction of GO, quercetin (Q)—a natural flavonoid—was used. To this end, before the reduction, the GO-padded fabrics were immersed in Q solutions in ethanol with different Q concentrations. Q enhanced the thermal reduction of GO. Depending on the Q concentration in the solutions, electrical surface resistivities of the cotton fabric of 750 kΩ/sq to 3.3 MΩ/sq and of the PET fabric of 240 kΩ/sq to 730 kΩ/sq were achieved. The cotton and PET fabrics also became hydrophobic, with water contact angles of 163° and 147°, respectively. In addition to the electrical conductivity, the presence of Q resulted in antibacterial activity of the fabrics against *Escherichia coli* and *Staphylococcus aureus*.

## 1. Introduction

Electrically conductive textiles are materials with a broad range of potential applications, from protective or smart cloth and wearable heaters to sensors, solar cells, and supercapacitors [1,2,3,4,5,6,7,8,9,10,11,12,13,14].

To impart electrical conductivity to synthetic polymers, they can be blended with conducting particles or nanoparticles. In turn, the deposition of conductive particles on fiber surfaces may impart electrical conductivity to natural fibers or ready-made synthetic fibers. Among others, carbon nanoparticles, including graphene and its derivatives (GM), are used for such a purpose [3,14,15,16]. The application of graphene for the modification of textiles is limited by the difficulty in obtaining stable aqueous dispersions of it, which can be applied for coating hydrophilic fibers, for instance, cotton. In turn, graphene oxide (GO) is hydrophilic and easily dispersible in water because of the presence of functional groups such as hydroxyl, carboxyl, carbonyl and epoxy groups. Epoxide and hydroxyl groups are located on the GO basal plane, whereas carboxyl groups are present on the edges of GO sheets [17]. GO has broad potential applications in biomedicine and nanomedicine because of its unique features, including a 2D planar structure, a large surface area, a high mechanical strength, ease of modification, chemical stability, and biocompatibility. However, GO’s functional groups have a negative effect on its electrical conductivity. To improve its conductivity, GO has to be reduced through thermal or chemical treatment to reduced graphene oxide (rGO) This reduction also increases the hydrophobicity and π-π stacking important for use in drug delivery [17,18].

The chemical reduction of GO to rGO requires an elevated temperature, 80–100 °C, and needs a relatively long time, from several hours to days. Thermal reduction demands an even higher temperature and an oxygen-free atmosphere is beneficial [19,20,21]. 

In our previous studies, the electrical conductivity of cotton fabric was achieved by coating it with GO and a subsequent thermal GO reduction by heating to 220 °C and short annealing at this temperature. To enhance the reduction, L-ascorbic acid and antioxidants applied in the plastic industry were used [22,23]. Depending on the antioxidant used, the fabric samples became hydrophilic or hydrophobic. It is also of importance that cotton decomposition occurs above 240–250 °C [24]. 

The growing interest in smart garments, which are electroconductive, sensitive to motion, and able to store energy or communicate, poses additional demands for the textiles used in such applications. In particular, antimicrobial activity is beneficial as it can limit or eradicate microbial colonization and the infection risk. The use of particles of GM offers such a possibility. However, the antibacterial activity of GM depends on many factors, such as the dispersibility, adsorption ability, number of corners and sharp edges [25], and is affected not only by particle shape, lateral size, and layer number but also by modifications of the surface, dispersion, and agglomeration. 

Three main mechanisms of antibacterial activity of GM are described in the literature. The main mechanisms of antibacterial activity of rGO are related to the nanoblade effect and oxidative stress [25]. The first one, frequently observed, is related to sharp particle edges, and termed as the nanoblade effect. The nanoblades, called also nanoknives or cutters, cut cell membranes and damage them, resulting in bacteria death [26,27,28]. The second mechanism is oxidative stress, caused by reactive oxygen species, leading to bacteria DNA damage and mitochondrial dysfunction [29]. The third mechanism, observed in solutions rather than in coatings, is the wrapping or trapping of bacteria by GM platelets due to their flexible structure, which isolates the bacteria from the environment [30]. In our previous studies, the antibacterial activity of GO and rGO coatings of cotton fabric was greatly limited [31]. The flakes, adhering to cotton fibers and lying flat on them, did not expose any sharp edges. Moreover, no wrapping or trapping of bacteria by the flakes was found. It is also worth noting that to impart electroconductivity to the fabric, the amount of rGO particles deposited on the fibers should be sufficient to create a 3D network, but the full coverage of fibers is not required, and the uncoated areas of cotton fibers were prone to bacteria colonization. 

In our previous work, to make the rGO-coated fabric antibacterial, Ag particles were deposited electrochemically [31]. Others have obtained electrically conductive and antibacterial rGO/Ag-modified linen and cotton fabrics by means of the simultaneous reduction of GO and silver nitrate with trisodium citrate [32]. To make poly(ethylene terephthalate) (PET) fabric electrically conductive and antibacterial, the simultaneous reduction of GO and silver nitrate was carried out with the use of ascorbic acid [33]. 

Recently, there has been a growing interest in the use of natural compounds in diverse fields, including the synthesis of nanoparticles [34] and reduction of GO [35,36], especially considering that among chemical reducing agents, the most efficient reductant is hydrazine, which is toxic and harmful to the environment. The use of natural reductants is especially important for the reduction of GO deposited on natural fibers, where the use of harmful compounds is excluded. Flavonoids, belonging to natural polyphenols, are antioxidants [37] and also exhibit antibacterial activity [38]. Quercetin (Q) is a natural flavonoid that occurs in plants, plant-derived foods, and nutritional supplement products. It exhibits anticancer, anti-inflammatory, antiallergy, antibacterial, and antiviral properties [39,40,41,42]. A novel drug delivery system based on GO loaded with Q was recently developed [43]. Q was also utilized to chemically reduce GO in order to cast Q-rGO on paraffin impregnated graphite electrodes for analysis of metal ions [44].

According to our knowledge, Q has not been used to reduce GO coatings on a textile. Being a natural flavonoid, Q is a promising eco-friendly reducing agent for GO deposited on fibers, including natural fibers. In the present study, Q was applied to assist the thermal reduction of GO deposited on cotton and PET fabrics. The fabrics were immersed in Q solutions in ethanol and heated to reduce the GO. This treatment allowed to achieve electrical conductivity of the fabrics, dependent on Q concentration in the solutions. Electrical surface resistivities of 750 kΩ/sq and 240 kΩ/sq for the cotton and PET fabrics, respectively, were reached. After the reduction, the fabrics were hydrophobic. Moreover, the presence of Q imparted antibacterial activity against *Escherichia coli* and *Staphylococcus aureus* to the fabrics.

## 2. Materials and Methods

### 2.1. Materials

White plain weave cotton fabric (145 g/m^2^) of 0.36 mm thickness with a weft of 205 threads/10 cm and a warp of 295 threads/10 cm was used in this study. Before further experiments, the fabric was cleaned by diethyl ether and anhydrous ethanol extraction, as previously described [45]. Also, plain weave PET fabric (Wistil, Kalisz, Poland) (89 g/m^2^) of 0.19 mm thickness was used, with a warp of 390 threads/10 cm, and a weft of 320 threads/10 cm.

A 1 wt.% aqueous dispersion of GO was supplied by NanoCarbon (Warsaw, Poland). According to the supplier, the I_D_/I_G_ value was >1.87 based on Raman spectroscopy. Elemental analysis evidenced the following element contents (%*w*/*w*): C, >45%; H, <2.5%; N, <0.5%; O, <49%; other, <4%. An impurity analysis by X-ray fluorescence spectrometry yielded: S (2.5%) > Ca (1%) > Mn (0.5%) > K (0.3%) > Cl (0.08%) = Fe (0.08%) > Cu (0.02%) = Zn (0.02%) > Ni (0.007%) > Cr (0.006%). Before further use, to reduce the GO concentration to 0.1 wt.%, the dispersion was diluted with distilled water. Then, it was subjected to sonication using a Sonopuls HD 2200 ultrasonic homogenizer (Bandelin, Berlin, Germany) at room temperature (RT) for 30 min (amplitude 40%, frequency 20 kHz).

Anhydrous Q (3,3′,4′,5,7-pentahydroxyflavone, C_15_H_10_O_7_), a flavonoid with a molar mass of 302.24 g/mol, was purchased from Apollo Scientific (Stockport, UK). Ethanol and diethyl ether were purchased from POCH (Gliwice, Poland) and CHEMPUR (Piekary Slaskie, Poland), respectively.

### 2.2. Modification of Fabrics

The fabrics were padded with the GO dispersion using a laboratory double-roll padding machine with horizontal squeezing rollers at a rate of 0.01 m/min, and then dried in the air at 100 °C for 15 min. The padding and drying cycle was repeated four times, as described previously in [22,23,45].

Strips of fabrics coated with GO were immersed in solutions of anhydrous Q in ethanol with concentrations of 0.25, 0.5, and 1 wt.% for 5 min, and then dried at RT. It should be noted that the solubility of Q in ethanol in room conditions is approx. 1 wt.% [46].

To reduce GO, the fabric strips, both strips treated with Q and untreated strips, placed between microscope glasses were thermally treated in a hot-stage FP82 equipped with an FP90 temperature controller from Mettler Toledo (Greifensee, Switzerland). They were heated from RT to 220 °C, annealed at 220 °C for 1 min, and then cooled down to RT at 10 °C/min. 

The obtained materials are referred to as PrGO, CrGO, PrGO/Q, and CrGO/Q, where P and C denote PET and cotton, respectively, and Q denotes Q-assisted reduction of GO. The number following the letter Q, for example, PrGO/Q0.5 or CrGO/Q0.5, indicates the percentage of Q in ethanol.

### 2.3. Characterization

Fabric samples, before and after modification, were vacuum-sputtered with 10 nm gold layers using a Quorum EMS150R ES (Laughton, UK) and examined with a scanning electron microscope (SEM), JEOL 6010LA (Tokyo, Japan). 

The thermal properties of the PET fabric before and after thermal treatment were studied using a DSC3 differential scanning calorimeter from Mettler Toledo (Greifensee, Switzerland) during heating at a rate of 10 °C/min to 300 °C in a nitrogen atmosphere.

The decrease in surface electrical resistivity (R) of the samples during the reduction was monitored via the 2-wire method, whereas after 24 h of stabilization under room conditions, R was determined via the 4-wire method using a Keithley 2400C SourceMeter (Cleveland, OH, USA). Copper electrodes were attached to the specimen surfaces at 1 cm apart with Dotite D-550 silver-containing paste (Fujikura Kasei, Tokyo, Japan). The results were averaged over at least three measurements of each material. Electrical impedance measurements were carried out at RT in a frequency range of 20 Hz–1 MHz by a Hewlett Packard 4284A Precision LCR Meter (Palo Alto, CA, USA). Two copper electrodes were attached to the specimen surface with silver paint at a distance of 1 cm apart. A 1 V amplitude of the sinusoidal voltage was maintained.

The water contact angles (WCAs) of the fabric samples were measured using the drop method with 5 μL drops at RT. A 100–00-230 NRL Rame Hart goniometer (Succasunna, NJ, USA) and the ImageJ Drop Analysis program (1.46r) were used for this purpose. The measurements were carried out five times and average WCA values were calculated.

The antibacterial properties of the fabric samples against the Gram-positive bacterial strain *Staphylococcus aureus* (*S. aureus*) ATCC 6538 and the Gram-negative bacterial strain *Escherichia coli* (*E. coli*) ATCC 8739 were analyzed according to Antibacterial Activity Assessment of Textile Materials: Agar Diffusion Test (ISO 20645:2004 [47]). At first, 15 mL portions of nutritive agar medium (Trypticase Soy Agar, Merck, Darmstadt, Germany) were poured onto sterile Petri dishes. The inoculum of a bacterial tested culture of 1–5 × 10^8^ CFU/mL (0.2 mL) was then poured onto the agar plates. Then, fabric specimens in the form of disks with a 6 mm diameter were placed on the agar surface. After 24 h incubation at 37 °C, the contact zones under the specimens were visually analyzed, and then the bacterial growth inhibition zones, if present, were measured. To evaluate the bacterial colonization, the disk specimens removed from the agar were also analyzed by SEM.

## 3. Results and Discussion

### 3.1. Graphene Oxide Reduction and Electrical Resistivity of Modified Fabrics

**Figure 1:**
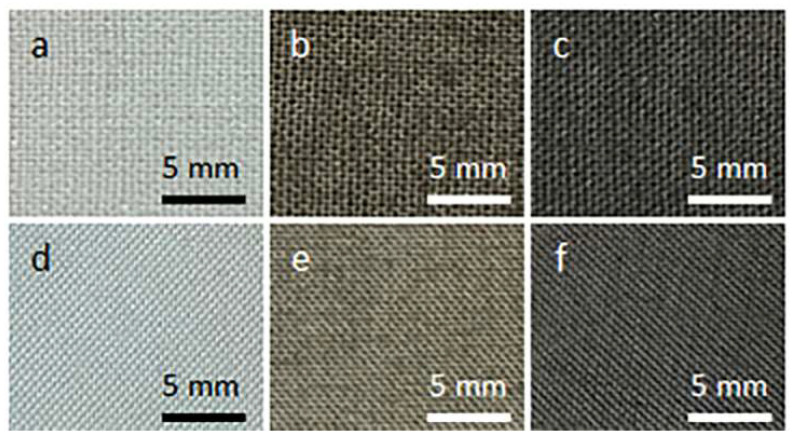
Cotton (**a**–**c**) and PET (**d**–**f**) fabric samples before coating with GO (**a**,**d**), after coating with GO (**b**,**e**), and after thermal reduction of GO (**c**,**f**).

Figure 1 shows cotton and PET fabric samples before and after coating with GO and after thermal reduction of GO. Coating with GO made the fabrics brown. After the thermal reduction of GO, the fabrics darkened.

**Figure 2:**
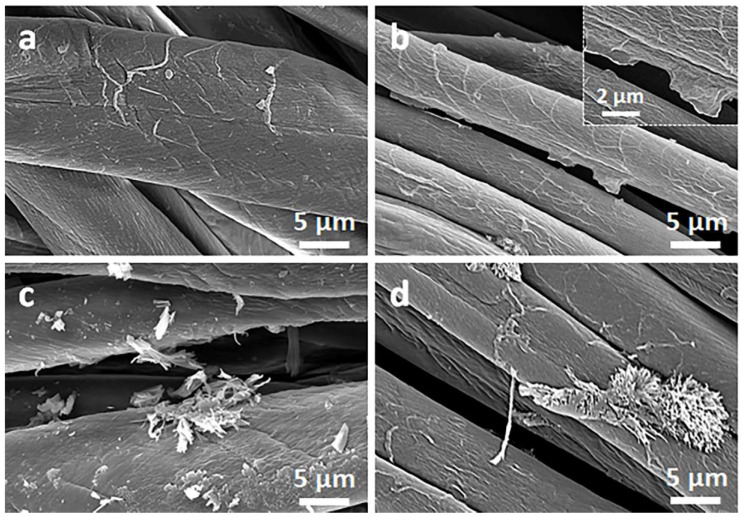
SEM micrographs of cotton fabric before (**a**) and after deposition of GO (**b**), after immersion in 1 wt.% solution of Q in ethanol (**c**), and after thermal reduction of GO (CrGO/Q1) (**d**).

**Figure 3:**
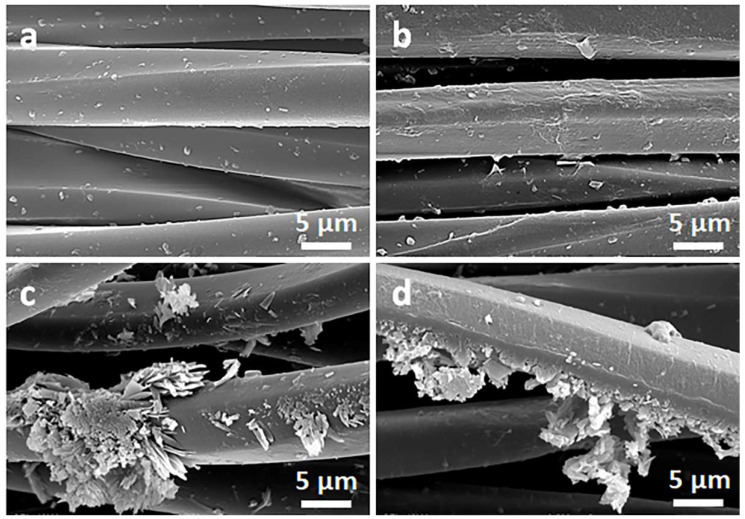
SEM micrographs of PET fabric before (**a**) and after deposition of GO (**b**), after immersion in 1 wt.% solution of Q in ethanol (**c**), and after thermal reduction of GO (PrGO/Q1) (**d**).

Figure 2 and Figure 3 show SEM micrographs of the cotton fabric and the PEF fabric, respectively, before and after deposition of GO, after immersion in 1 wt.% solution of Q in ethanol, and after thermal reduction of GO.

Due to their small thickness and good dispersion, GO or rGO flakes were only occasionally discernible on the rough cotton fibers, as shown in the inset in Figure 2b. The flakes were better seen on smooth PET fibers, as illustrated in Figure 3b.

On the contrary, on the fibers immersed in a Q solution in ethanol, Q crystals were easily observed before and after the thermal reduction of GO, as illustrated in Figure 2c,d and Figure 3c,d. It is worth noting that the thermal treatment did not alter the cotton and PET fiber surface morphology.

**Figure 4:**
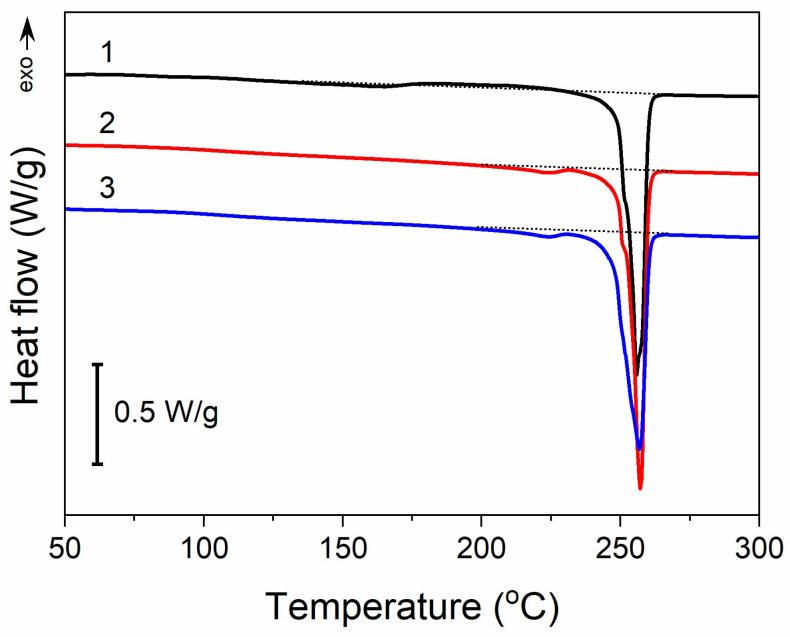
DSC heating thermograms of PET fabric before thermal treatment (1), after thermal treatment analogous to that during thermal reduction of GO (2), and (3) after thermal reduction of GO (PrGO/Q1). Heating rate of 10 °C/min.

Moreover, thermal treatment did not induce any significant changes in the thermal properties of PET fabric, as illustrated in Figure 4. The heating thermogram of the untreated PET fabric exhibited a melting peak at 255 °C with an enthalpy of 57 J/g. In addition, it exhibited a very weak and broad melting endotherm followed by a cold crystallization exotherm with extrema at 163 °C and 208 °C, respectively. The small enthalpies associated with these transitions, about 3–4 J/g, were similar. After the thermal treatment analogous to that used for GO reduction, and also after Q-assisted rGO reduction (PrGO/Q1), a low-temperature endotherm peak appeared at 224 °C, with an enthalpy of 1.5 J/g, whereas cold crystallization was absent. However, the main melting peak temperature remained unchanged, and its enthalpy only insignificantly decreased by 2–3 J/g. Thus, the crystallinity of PET, calculated assuming the heat of fusion of crystals is 133–140 J/g [48,49], remained practically the same at near 40%. 

**Figure 5:**
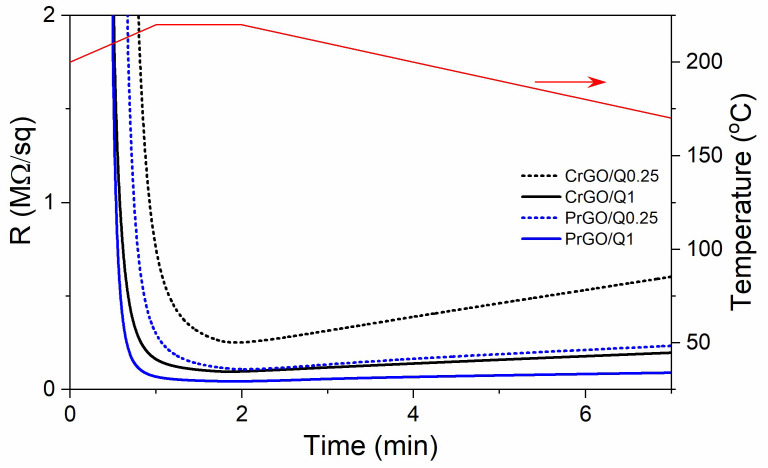
Exemplary dependencies of electrical surface resistivity, R, of the GO-coated cotton and PET fabric on time during Q-assisted thermal reduction of GO (black and blue lines) and temperature dependence on time (red line).

**Figure 6:**
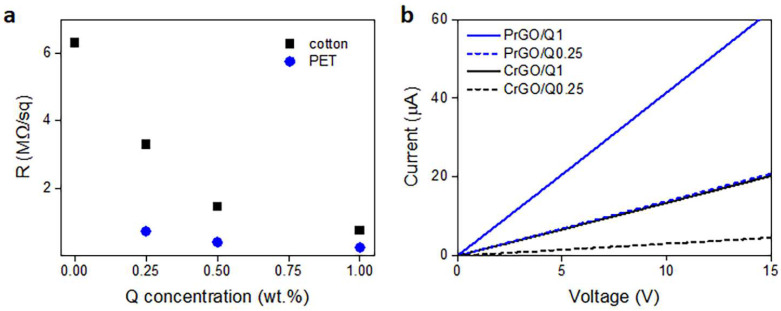
Electrical surface resistivity, R, of cotton and PET fabrics after thermal reduction of GO depending on Q concentration in its solution in ethanol (**a**), and exemplary current–voltage characteristics measured 24 h after thermal reduction of GO (**b**).

During heating and annealing at 220 °C, the R of GO-coated fabrics decreased due to the reduction of GO as shown in Figure 5. However, R increased during cooling and further holding at RT, but stabilized after 24 h. The resistivity of GM particles is known to increase with decreasing temperatures [50]; however, such a dependence could not be responsible for the several-fold increase in R of the fabrics, especially considering that the increase also occurred at RT. Such an increase in R was observed and discussed by us previously [22], and was attributed to the partial reversibility of the reduction. The values of R of the fabrics measured 24 h after the reduction are shown in Figure 6a and Table 1. The R values of the fabrics untreated with Q were the highest, and that of PrGO was above the measurement range of the equipment, 50 MΩ. The current–voltage characteristics of all samples, shown in Figure 6b, were linear, evidencing that Ohm’s law was satisfied. 

Cellulose is a polymer composed of d-glucose units linked through covalent β-1,4- glycosidic bonds. We hypothesize that the glucose units could assist the thermal reduction of GO, as glucose is a mild reducing agent for GO and was recently used to synthesize glucose-rGO-supported Ag-Cu_2_O nanocomposites [51]. The application of Q to assist the reduction of GO permitted a reduction in the R of the fabrics; it decreased with increasing concentrations of Q in ethanol solutions, as shown in Figure 6a, although the dependence was weaker for larger Q concentrations. At 1 wt.% Q concentration, an R of 750 kΩ/sq and 240 kΩ/sq was achieved for cotton (CrGO/Q1) and PET (PrGO/Q1) fabrics, respectively. However, the R values of CrGO/Q were higher than those of PrGO/Q. We hypothesize that after the strongly enhanced reduction of GO, the surface morphology of the fibers becomes an important factor. The surface roughness of cotton fibers, illustrated in Figure 2, could negatively affect the contacts between rGO platelets and this could result in a higher resistance of the rGO-conducting network on fiber surfaces. It is worth mentioning that an adverse effect of polylactide surface roughness on the formation of a conducting network of multi-wall carbon nanotubes (MWCNTs) was observed by us previously [52].

**Figure 7:**
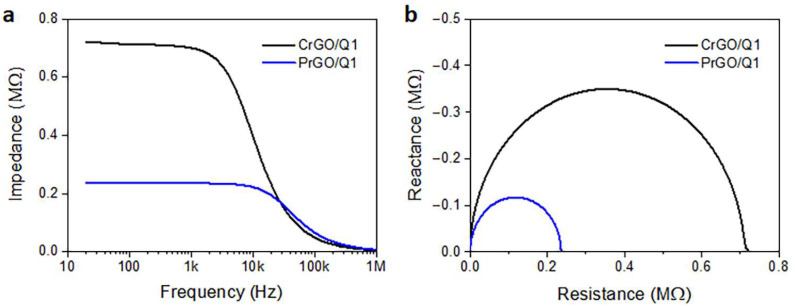
Electrical impedance modulus of CrGO/Q1 and PrGO/Q1 (**a**) and Nyquist plots of reactance vs. resistance (**b**).

Frequency dependencies of the electrical impedance modulus of CrGO/Q1 and PrGO/Q1 are plotted in Figure 7a. The corresponding Nyquist plots of the real and imaginary parts of the complex impedances are shown in Figure 7b. They are in the form of semicircular arcs, with diameters corresponding to the fabric resistances. In a broad frequency range, the fabric impedances were dominated by their frequency-independent resistances, whose values corresponded to R values determined independently in DC experiments. At higher frequencies, transitions to a frequency-dependent regime dominated by a negative reactance, that is, capacitance, were observed. For the material with a higher low-frequency impedance and R, the frequency at which the transition occurred was lower. Similar behavior was observed by us previously [52] for polylactide nonwovens modified with MWCNTs. The frequency-independent impedance was interpreted as being determined by direct contacts between the MWCNTs forming the conducting network. The impedance spectroscopy results confirmed the dominant role of direct rGO contacts on the electrical properties of rGO-modified fabrics in a broad frequency range.

**Figure 8:**
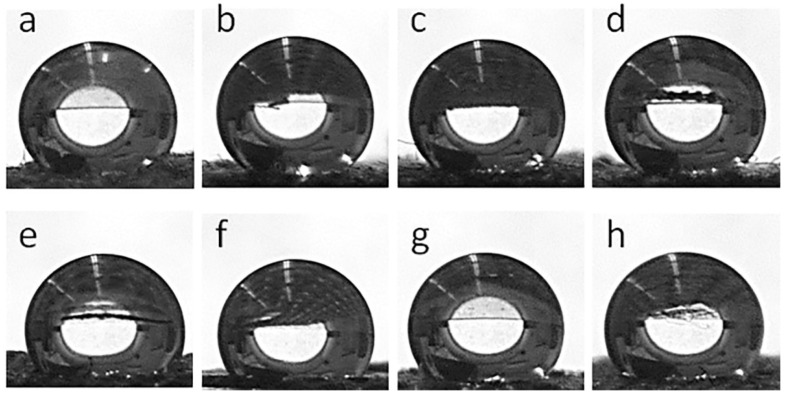
Water droplets deposited on fabric surfaces: CrGO (**a**), CrGO/Q0.25 (**b**), CrGO/Q0.5 (**c**), CrGO/Q1 (**d**), PrGO (**e**), PrGO/Q0.25 (**f**), PrGO/Q0.5 (**g**), and PrGO/Q1 (**h**).

Figure 8 shows water droplets on the fabric surfaces, whereas the corresponding WCA values are collected in Table 1. Both the cotton and PEF fabrics were hydrophilic and water droplets soaked into them, although this was slower in the PET fabric than in the cotton fabric. The GO-coated fabrics behaved similarly. However, after the thermal reduction of GO, all fabric samples became hydrophobic. WCAs of 155° and 144° were measured for CrGO and PrGO, respectively, which increased to 163° and 147° for CrGO/Q and PrGO/Q. It seems that the surface roughness of cotton fibers, which adversely affected the R of CrGO/Q, improved their hydrophobicity, as the air trapped in pockets and between protrusions of rGO-coated cotton fibers could enhance the hydrophobicity of the fabric [53].

### 3.2. Antibacterial Properties

**Figure 9:**
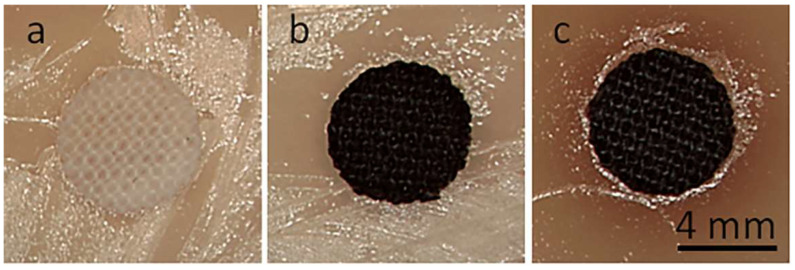
Cotton fabric disks with a 6 mm diameter in *S. aureus*-inoculated agar after 24 h of incubation: neat cotton (**a**), CrGO (**b**), CrGO/Q1 (**c**).

**Figure 10:**
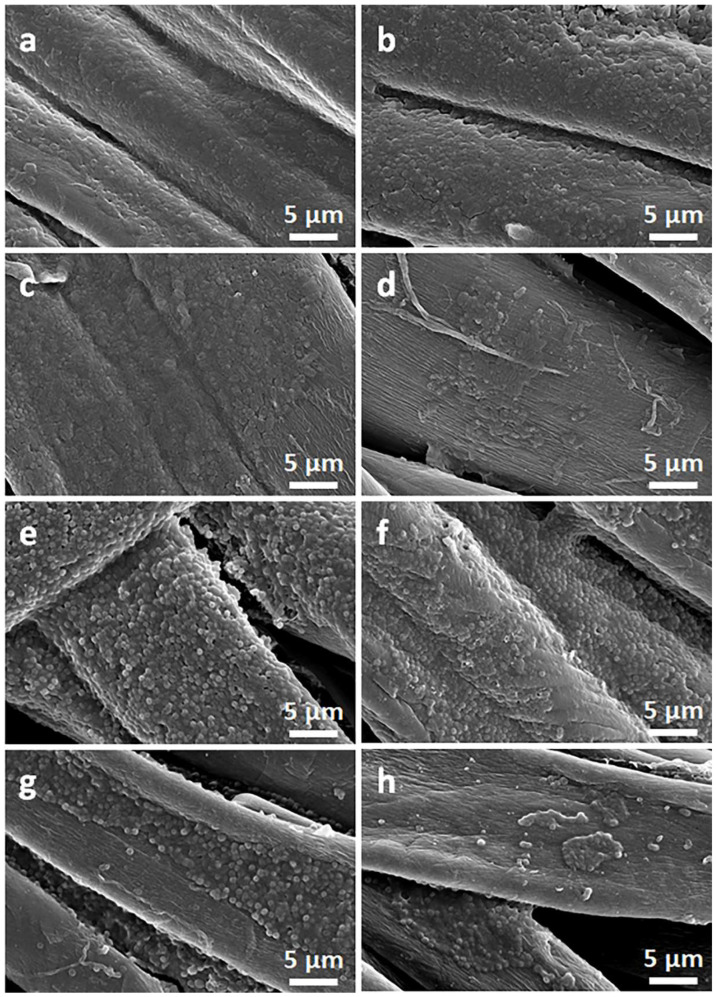
SEM micrographs of bottom sides of cotton fabric specimens removed from *E. coli* (**a**–**d**) and *S. aureus* (**e**–**h**) inoculated agar: pure cotton (**a**,**e**), CrGO (**b**,**f**), CrGO/Q0.25 (**c**,**g**), and CrGO/Q1 (**d**,**h**).

**Figure 11:**
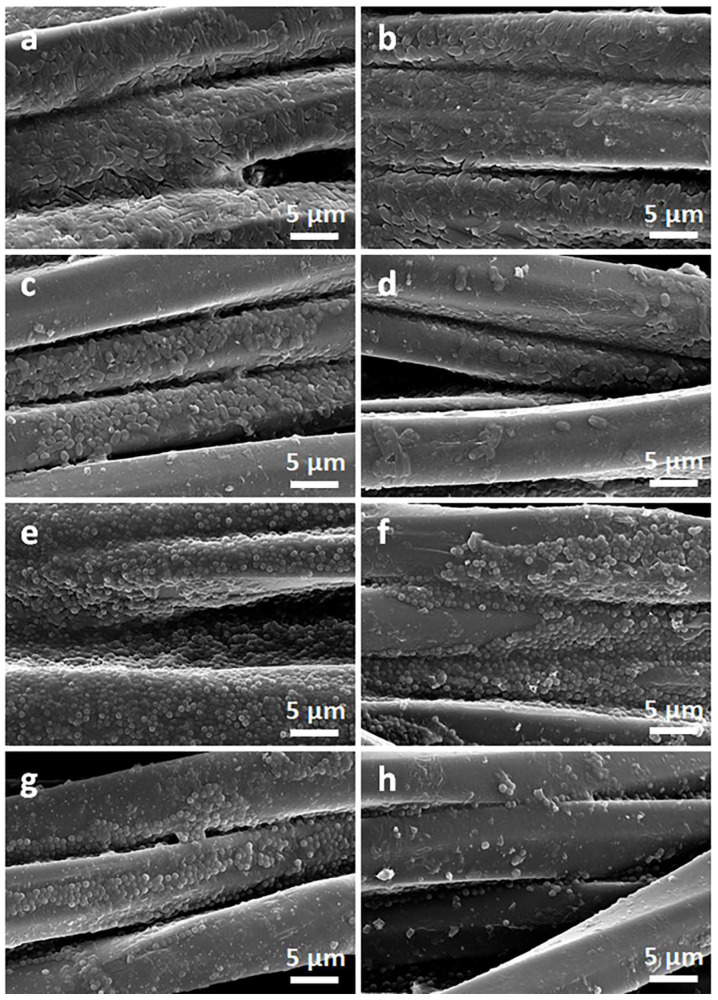
SEM micrographs of bottom sides of PET fabric specimens removed from *E. coli* (**a**–**d**) and *S. aureus* (**e**–**h**) inoculated agar: pure PET (**a**,**e**), PrGO (**b**,**f**), PrGO/Q0.25 (**c**,**g**), and PrGO/Q1 (**d**,**h**).

Exemplary photographs of the fabric specimens lying on inoculated agar after bacteria incubation are shown in Figure 9. Bacterial growth inhibition zones were barely noticeable, but a clearly reduced growth close to the tested materials was visible. The zone was only larger around CrGO/Q1 on *S. aureus*-inoculated agar, with a diameter of 6.6 mm. However, the absence of bacterial growth inhibition zones around specimens does not exclude their antibacterial activity in direct contact with microorganisms. The antibacterial activity of the CrGO/Q and PrGO/Q coated fabric specimens was corroborated by SEM analysis of their surfaces, which were in contact with the inoculated agar. Both cotton and PET fabrics, shown in Figure 10 and Figure 11, were overgrown with *E. coli* and *S. aureus*. The same applies to the fabrics coated with GO. It seems that slightly less bacteria were seen on CrGO and PrGO, most possibly due to the hydrophobicity of these materials, which made the adhesion of bacteria more difficult. However, the bacterial colonization was significantly reduced only on CrGO/Q and PrGO/Q. The amount of bacteria seen on CrGO/Q0.25 and PrGO/Q0.25 was significantly decreased in comparison to that on the materials untreated with Q, as shown in Figure 10c,g and Figure 11c,g. Similar results were obtained for CrGO/Q0.5 and PrGO/Q0.5. However, the strongest antibacterial activity was evidenced for CrGO/Q1 and PrGO/Q1, on which the bacterial growth was strongly reduced. Moreover, many of these bacterial cells were flattened and damaged. Such changes have been attributed by others to bacteria damage and death caused by contact with antimicrobial materials [54,55,56]. 

It is worth noting that Q decomposes during heating. According to [57], the first stage of decomposition in air occurs between 103 and 342 °C, although mass loss in this temperature range is only 10%. The obtained results evidenced that despite its minor decomposition, the antibacterial activity of Q did not vanish after the thermal treatment and reduction of GO.

## 4. Conclusions

Cotton and PET fabrics were coated with a GO dispersion using the padding method, and then GO was reduced thermally to rGO with the assistance of quercetin, Q, a natural flavonoid. The use of Q enhanced the reduction of GO, leading to a diminished electrical surface resistivity of the materials, R. The R decreased with increasing concentration of Q in an ethanol solution, in which the GO-coated fabrics were immersed before the thermal reduction of GO. At 1 wt.% Q concentration, an R of 750 kΩ/sq and 240 kΩ/sq was achieved for cotton and PET fabric, respectively. The higher R of cotton fabrics was most probably related to the roughness of cotton fibers having an adverse effect on the formation of the conducting network of rGO platelets on fiber surfaces. After the reduction of the GO deposited on the fibers, both cotton and PET fabrics became hydrophobic. Q-assisted reduction of GO resulted in WCA values of 163° and 147°, respectively. Moreover, treatment with Q resulted in antibacterial activity of the materials against *E. coli* and *S. aureus*. SEM analysis proved the reduced bacteria colonization on all Q-treated fibers. The strongest effect was evidenced for the fabrics immersed in a 1 wt.% solution of Q in ethanol before GO reduction, which agreed with the presence of bacterial growth inhibition zone for CrGO/Q1. The combination of electrical conductivity, hydrophobicity, and antibacterial activity makes the cotton and PET fabrics coated with rGO obtained by Q-assisted reduction of GO interesting materials for a broad range of applications.

## Figures and Tables

**Table 1:** Electrical surface resistivity, R, and water contact angles, WCAs, of cotton and PET fabrics after thermal reduction of GO, depending on Q concentration in solution in ethanol.

Concentration of Q (%)	Cotton		PET	
	R (MΩ/sq)	WCA (°)	R (MΩ/sq)	WCA (°)
-	6.3	155	>50	144
0.25	3.3	163	0.73	146
0.5	1.4	163	0.40	147
1	0.75	163	0.24	147

## Data Availability

The data are available on request from the corresponding authors.

## References

[1] Islam G.M.N., Ali A., Collie S. (2020). Textile sensors for wearable applications: A comprehensive review. Cellulose.

[2] Maity S., Chatterjee A. (2018). Conductive polymer-based electro-conductive textile composites for electromagnetic interference shielding: A review. J. Ind. Text..

[3] Molina J. (2016). Graphene-based fabrics and their applications: A review. RSC Adv..

[4] Stoppa M., Chiolerio A. (2014). Wearable Electronics and Smart Textiles: A Critical Review. Sensors.

[5] Kowalczyk D., Fortuniak W., Mizerska U., Kaminska I., Makowski T., Brzezinski S., Piorkowska E. (2017). Modification of cotton fabric with graphene and reduced graphene oxide using sol-gel method. Cellulose.

[6] Wang D., Li D., Zhao M., Xu Y., Wei Q. (2018). Multifunctional wearable smart device based on conductive reduced graphene oxide/polyester fabric. Appl. Surf. Sci..

[7] Seyedin S., Razal J.M., Innis P.C., Jeiranikhameneh A., Beirne S., Wallace G.G. (2015). Knitted Strain Sensor Textiles of Highly Conductive All-Polymeric Fibers. ACS Appl. Mater. Interfaces.

[8] Zahid M., Papadopoulou E.L., Athanassiou A., Bayer I.S. (2017). Strain-responsive mercerized conductive cotton fabrics based on PEDOT:PSS/graphene. Mater. Design.

[9] Hong S., Lee H., Lee J., Kwon J., Han S., Suh Y.D., Cho H., Shin J., Yeo J., Ko S.H. (2015). Highly Stretchable and Transparent Metal Nanowire Heater for Wearable Electronics Applications. Adv. Mater..

[10] Jost K., Perez C.R., McDonough J.K., Presser V., Heon M., Dion G., Gogotsi Y. (2011). Carbon coated textiles for flexible energy storage. Energy Environ. Sci..

[11] Attia N.F., El Ebissy A.A., Hassan M.A. (2015). Novel synthesis and characterization of conductive and flame retardant textile fabrics. Polym. Adv. Technol..

[12] Nooralian Z., Parvinzadeh Gashti M., Ebrahimi I. (2016). Fabrication of a multifunctional graphene/polyvinylphosphonic acid/cotton nanocomposite via facile spray layer-by-layer assembly. RSC Adv..

[13] Huang Y., Zhu M., Pei Z., Xue Q., Huang Y., Zhi C. (2016). A shape memory supercapacitor and its application in smart energy storage textiles. J. Mater. Chem. A.

[14] Sahito I.A., Sun K.C., Arbab A.A., Qadir M.B., Choi Y.S., Jeong S.H. (2016). Flexible and conductive cotton fabric counter electrode coated with graphene nanosheets for high efficiency dye sensitized solar cell. J. Power Sources.

[15] Makowski T., Zhang C., Olah A., Piorkowska E., Baer E., Kregiel D. (2019). Modification of dual-component fibrous materials with carbon nanotubes and methyltrichlorosilane. Mater. Design.

[16] Shateri-Khalilabad M., Yazdanshenas M.E. (2013). Fabricating electroconductive cotton textiles using graphene. Carbohydr. Polym..

[17] Bellier N., Baipaywad P., Ryu N., Lee J.Y., Park H. (2022). Recent biomedical advancements in graphene oxide- and reduced graphene oxide-based nanocomposite nanocarriers. Biomater. Res..

[18] Guex L.G., Sacchi B., Peuvot K.F., Andersson R.L., Pourrahimi A.M., Ström V., Farris S., Olsson R.T. (2017). Experimental review: Chemical reduction of graphene oxide (GO) to reduced graphene oxide (rGO) by aqueous chemistry. Nanoscale.

[19] Chua C.K., Pumera M. (2014). Chemical reduction of graphene oxide: A synthetic chemistry viewpoint. Chem. Soc. Rev..

[20] Pei S., Cheng H.-M. (2012). The reduction of graphene oxide. Carbon.

[21] Huh S.H., Mikhailov S. (2011). Thermal Reduction of Graphene Oxide. Physics and Applications of Graphene.

[22] Makowski T., Svyntkivska M., Piorkowska E., Mizerska U., Fortuniak W., Kowalczyk D., Brzezinski S. (2018). Conductive and superhydrophobic cotton fabric through pentaerythritol tetrakis(3-(3,5-di-tert-butyl-4-hydroxyphenyl)propionate) assisted thermal reduction of graphene oxide and modification with methyltrichlorosilane. Cellulose.

[23] Jedrzejczyk M., Makowski T., Svyntkivska M., Piorkowska E., Mizerska U., Fortuniak W., Brzezinski S., Kowalczyk D. (2019). Conductive cotton fabric through thermal reduction of graphene oxide enhanced by commercial antioxidants used in the plastics industry. Cellulose.

[24] Matheus P., Vinícios P., Ademir J.Z., van de Ven T., Godbout L. (2013). Structural Characteristics and Thermal Properties of Native Cellulose. Cellulose.

[25] Cao G., Yan J., Ning X., Zhang Q., Wu Q., Bi L., Zhang Y., Han Y., Guo J. (2021). Antibacterial and antibiofilm properties of graphene and its derivatives. Colloid Surf. B-Biointerfaces.

[26] Nasirzadeh N., Azari M.R., Rasoulzadeh Y., Mohammadian Y. (2019). An assessment of the cytotoxic effects of graphene nanoparticles on the epithelial cells of the human lung. Toxicol. Ind. Health.

[27] Pulingam T., Thong K.L., Appaturi J.N., Nordin N.I., Dinshaw I.J., Lai C.W., Leo B.F. (2020). Synergistic antibacterial actions of graphene oxide and antibiotics towards bacteria and the toxicological effects of graphene oxide on human epidermal keratinocytes. Eur. J. Pharm. Sci..

[28] Zainal-Abidin M.H., Hayyan M., Ngoh G.C., Wong W.F. (2019). From nanoengineering to nanomedicine: A facile route to enhance biocompatibility of graphene as a potential nano-carrier for targeted drug delivery using natural deep eutectic solvents. Chem. Eng. Sci..

[29] Khan B., Adeleye A.S., Burgess R.M., Russo S.M., Ho K.T. (2019). Effects of graphene oxide nanomaterial exposures on the marine bivalve, Crassostrea virginica. Aquat. Toxicol..

[30] Akhavan O., Ghaderi E., Esfandiar A. (2011). Wrapping Bacteria by Graphene Nanosheets for Isolation from Environment, Reactivation by Sonication, and Inactivation by Near-Infrared Irradiation. J. Phys. Chem. B.

[31] Makowski T., Svyntkivska M., Piorkowska E., Mizerska U., Fortuniak W., Kowalczyk D., Brzezinski S., Kregiel D. (2022). Antibacterial Electroconductive Composite Coating of Cotton Fabric. Materials.

[32] Farouk A., Saeed S.E.-S., Sharaf S., Abd El-Hady M.M. (2020). Photocatalytic activity and antibacterial properties of linen fabric using reduced graphene oxide/silver nanocomposite. RSC Adv..

[33] Moazami A., Montazer M., Dolatabadi M.K. (2016). Tunable functional properties on polyester fabric using simultaneous green reduction of graphene oxide and silver nitrate. Fiber. Polym..

[34] Mirza K., Naaz F., Ahmad T., Manzoor N., Sardar M. (2023). Development of Cost-Effective, Ecofriendly Selenium Nanoparticle-Functionalized Cotton Fabric for Antimicrobial and Antibiofilm Activity. Fermentation.

[35] Manchala S., Tandava V.S.R.K., Jampaiah D., Bhargava S.K., Shanker V. (2019). Novel and Highly Efficient Strategy for the Green Synthesis of Soluble Graphene by Aqueous Polyphenol Extracts of Eucalyptus Bark and Its Applications in High-Performance Supercapacitors. ACS Sustain. Chem. Eng..

[36] De Silva K.K.H., Huang H.H., Joshi R.K., Yoshimura M. (2017). Chemical reduction of graphene oxide using green reductants. Carbon.

[37] Abbas M., Saeed F., Anjum F.M., Afzaal M., Tufail T., Bashir M.S., Ishtiaq A., Hussain S., Suleria H.A.R. (2017). Natural polyphenols: An overview. Int. J. Food Prop..

[38] Shamsudin N.F., Ahmed Q.U., Mahmood S., Ali Shah S.A., Khatib A., Mukhtar S., Alsharif M.A., Parveen H., Zakaria Z.A. (2022). Antibacterial Effects of Flavonoids and Their Structure-Activity Relationship Study: A Comparative Interpretation. Molecules.

[39] Kobylińska A., Janas K.M. (2015). Prozdrowotna rola kwercetyny obecnej w diecie człowieka—Health—Promoting effect of quercetin in human diet. Postepy Hig. Med. Dosw..

[40] Kobylińska A. (2017). Exogenous quercetin as a proliferation stimulator in tobacco BY-2 cells. J. Elem..

[41] Gomathi K., Gopinath D., Rafiuddin Ahmed M., Jayakumar R. (2003). Quercetin incorporated collagen matrices for dermal wound healing processes in rat. Biomaterials.

[42] Kost B., Svyntkivska M., Brzeziński M., Makowski T., Piorkowska E., Rajkowska K., Kunicka-Styczyńska A., Biela T. (2020). PLA/β-CD-based fibres loaded with quercetin as potential antibacterial dressing materials. Colloid Surf. B-Biointerfaces.

[43] Croitoru A.-M., Moroșan A., Tihăuan B., Oprea O., Motelică L., Trușcă R., Nicoară A.I., Popescu R.-C., Savu D., Mihăiescu D.E. (2022). Novel Graphene Oxide/Quercetin and Graphene Oxide/Juglone Nanostructured Platforms as Effective Drug Delivery Systems with Biomedical Applications. Nanomaterials.

[44] Krishna Kumar K., Devendiran M., Senthil Kumar P., Sriman Narayanan S. (2021). Quercetin-rGO based mercury-free electrode for the determination of toxic Cd (II) and Pb (II) ions using DPASV technique. Environ. Res..

[45] Makowski T., Kowalczyk D., Fortuniak W., Jeziorska D., Brzezinski S., Tracz A. (2014). Superhydrophobic properties of cotton woven fabrics with conducting 3D networks of multiwall carbon nanotubes, MWCNTs. Cellulose.

[46] Razmara R.S., Daneshfar A., Sahraei R. (2010). Solubility of Quercetin in Water + Methanol and Water + Ethanol from (292.8 to 333.8) K. J. Chem. Eng. Data.

[47] (2004). Textile Fabrics—Determination of Antibacterial Activity—Agar Diffusion Plate Test.

[48] Smith C.W., Dole M. (1956). Specific heat of synthetic high polymers. VII. Polyethylene terephthalate. J. Polym. Sci..

[49] Mehta A., Gaur U., Wunderlich B. (1978). Equilibrium melting parameters of poly(ethylene terephthalate). J. Polym. Sci. Polym. Phys. Ed..

[50] Sibilia S., Bertocchi F., Chiodini S., Cristiano F., Ferrigno L., Giovinco G., Maffucci A. (2021). Temperature-dependent electrical resistivity of macroscopic graphene nanoplatelet strips. Nanotechnology.

[51] Sharma K., Maiti K., Kim N.H., Hui D., Lee J.H. (2018). Green synthesis of glucose-reduced graphene oxide supported Ag-Cu_2_O nanocomposites for the enhanced visible-light photocatalytic activity. Compos. B Eng..

[52] Svyntkivska M., Makowski T., Shkyliuk I., Piorkowska E. (2023). Electrically conductive crystalline polylactide nonwovens obtained by electrospinning and modification with multiwall carbon nanotubes. Int. J. Biol. Macromol..

[53] Cassie A.B.D., Baxter S. (1944). Wettability of porous surfaces. Trans. Faraday Soc..

[54] Mei L., Zhang X., Wang Y., Zhang W., Lu Z., Luo Y., Zhao Y., Li C. (2014). Multivalent polymer—Au nanocomposites with cationic surfaces displaying enhanced antimicrobial activity. Polym. Chem..

[55] Chen R.-B., Zhang K., Zhang H., Gao C.-Y., Li C.-L. (2018). Analysis of the antimicrobial mechanism of porcine beta defensin 2 against *E. coli* by electron microscopy and differentially expressed genes. Sci. Rep..

[56] Shen J., Tian Y., Li Y., Ma R., Zhang Q., Zhang J., Fang J. (2016). Bactericidal Effects against S. aureus and Physicochemical Properties of Plasma Activated Water stored at different temperatures. Sci. Rep..

[57] da Costa E.M., Filho J.M.B., do Nascimento T.G., Macêdo R.O. (2002). Thermal characterization of the quercetin and rutin flavonoids. Thermochim. Acta.

